# The Effectiveness of a Cell Phone eHealth App in Changing Knowledge, Stigmatizing Attitudes, and Intention to Seek Help Associated With Obsessive-Compulsive Disorder: Pilot Questionnaire Study

**DOI:** 10.2196/48027

**Published:** 2024-03-29

**Authors:** Antonio Chaves, Sandra Arnáez, Gemma García-Soriano

**Affiliations:** 1 Departamento de Orientación Educativa IES Cid Campeador Conselleria d'Educació, Cultura i Esport Valencia Spain; 2 Departamento de Personalidad, Evaluación y Tratamientos Psicológicos Universitat de València Valencia Spain

**Keywords:** obsessive-compulsive disorder, OCD, mental health literacy, stigma, app, help-seeking intention, seek help, mobile phone

## Abstract

**Background:**

Obsessive-compulsive disorder (OCD) is a disabling disorder associated with high interference in people’s lives. However, patients with OCD either do not seek help or delay seeking help. Research suggests that this could be explained by poor mental health literacy about the disorder and the associated stigma.

**Objective:**

This study aims to evaluate the feasibility, acceptability, and preliminary effectiveness of a mental health mobile app, esTOCma, developed to improve knowledge about OCD and its treatment, increase help-seeking intention, and reduce stigmatizing attitudes and social distance associated with OCD.

**Methods:**

We used preintervention, postintervention, and 3-month follow-up assessments in this single-arm pilot intervention. Overall, 90 participants were recruited from the community using the snowball sampling method. We used esTOCma to defeat the “stigma monster” over the course of 10 missions. The participants completed the sociodemographic information and Obsessive-Compulsive Inventory–Revised at preassessment and an acceptability questionnaire at postassessment. All other measures were completed at the preassessment, postassessment, and 3-month follow-up (ie, the Spanish Mental Illness Stigma Attribution Questionnaire–27, the General Help-Seeking Questionnaire, the Social Distance Scale, and the Mental Health Literacy Questionnaire).

**Results:**

Of the 90 participants from the community that were assessed for eligibility, 86% (n=78) were allocated to intervention. Of these 78 participants, 79% (n=62) completed the game and answered the postintervention assessment (completer group). Overall, 69% (43/62) of the participants also completed the 3-month follow-up assessment. The participants completing the study were older (*P*=.003) and had a higher baseline knowledge of OCD (*P*=.05). The participants took an average of 13.64 (SD 10.50) days to complete the intervention, including the pre- and postassessments. The participants spent an average of 4.56 (SD 3.33) days completing the 10 missions included in the app. Each mission took a mean of between 2 (SD 3.01) and 9.35 (SD 3.06) minutes. The app was rated as useful or very useful by the vast majority of participants 90% (56/62). Moreover, 90% (56/62) of the participants reported that they had learned or learned a lot, and 98% (61/62) of the participants reported that they would recommend the app to a friend. Repeated measures ANOVA (43/62, 69%) showed that after the intervention participants showed an increased knowledge of mental health and intention to seek help as well as fewer stigmatizing attitudes and less social distance.

**Conclusions:**

Preliminary data show that esTOCma is a feasible and acceptable app, and after completing its 10 missions, there is an increase in the understanding of OCD and help-seeking intention along with a decrease in the social stigma and social distance associated with OCD that lasts for at least 3 months. The results support the potential of technology-based interventions to increase the intention to seek help and reduce the stigma associated with OCD. A larger, community-controlled study is also recommended.

## Introduction

### Background

Obsessive-compulsive disorder (OCD) is a clinically heterogeneous condition characterized by obsessions, compulsions or both that cause clinically significant levels of distress or functional impairment [1,2]. At present, effective treatments exist for OCD [3,4]; however, many people delay seeking treatment [5,6], and this may contribute to its chronic course [7,8]. Research suggests that this delay in seeking treatment by patients with OCD may be explained by social stigma and poor mental health literacy (MHL) about the disorder [5,9,10].

To date, many interventions have proved to be effective in reducing stigma associated with mental disorders, producing knowledge, and achieving attitudinal improvements [11-13]. Data show that contact and education strategies produce small-to-medium short-term reductions in stigmatizing attitudes, and there is limited evidence on long-term effectiveness [14,15]. A few of these interventions have benefited from the advantages of new technologies (eg, video games and electronic contact with patients), showing medium effects on reducing social stigma and suggesting that new technologies are a useful tool to decrease stigmatizing attitudes toward mental disorders, at least in the young population (the mean age of participants ranged between 15.7 and 24 years) [16]. However, none of these studies have examined the benefits of using app-based interventions. Furthermore, most of these interventions have focused on reducing the stigma of mental disorders such as schizophrenia [17-19], depression [20-22] or bipolar disorder [23,24].

In the case of OCD, programs are scarce, and most proposals have studied the impact of the educational mechanism [25-28], showing a reduction in stigmatizing attitudes and an improvement in participants’ MHL. Furthermore, a proposal has reported a significant reduction in social stigma and social distance using an indirect contact strategy through a video of a patient with OCD and a family member talking about their experience with the problem [29]. However, none of these interventions are based on innovative technologies, and only 1 of them includes >1 intervention strategy (ie, contact and education) [29]. In this context, as a response to the limited interventions focused on OCD and the need to bridge the gap between the onset of symptoms and seeking help, a gamified mental health app named esTOCma has been developed [30] (refer to the Methods section). In this way, the intervention will take advantage of mobile mental health interventions as low-cost tools that are available 24 hours a day for a large number of people [31,32], in addition to including gaming benefits such as providing immediate feedback, motivating users to achieve goals, and being easy to use [33].

### Objective and Hypothesis

The aim of this study is to explore the feasibility, acceptability, and preliminary effectiveness of the beta version of the eHealth mobile app esTOCma. Regarding the effectiveness, and based on the reviewed literature on interventions to reduce stigma and increase knowledge of OCD [25-29] and other mental disorders [11,14,16], we hypothesize that the esTOCma intervention will (1) improve knowledge associated with OCD and its treatment, (2) decrease stigmatizing attitudes and social distance, and (3) increase intention to seek help. Moreover, we hypothesize that changes will be maintained at the 3-month follow-up assessment.

## Methods

### Study Design

This study was a single-arm pilot intervention with 3 measures at the pretest, posttest, and 3-month follow-up. Data were collected from people residing in Spain.

### Participants and Procedure

Data were collected from a convenience sample. Participants were recruited from the general community and university setting by snowball sampling after providing relevant information via face-to-face classes and inviting the participants to share information about the study with their acquaintances. The inclusion criteria for this study were as follows: (1) being aged >18 years, (2) residing in Spain, (3) owning a smartphone, and (4) self-reporting not having an OCD diagnosis. Interested participants were invited to participate in a study consisting of downloading an app (esTOCma) in an Android Package Kit file format, playing with it, and completing a set of questionnaires before and after using the app. The participants performed all the tasks individually at home and at their own pace at the time they deemed most convenient. Furthermore, they chose the rate at which they completed the game, although the app recommends completing 1 mission a day.

The participants signing the informed consent form were given an identification number automatically generated by the app. The data from the questionnaires and the game were matched with the personal player ID. The participants were randomized by the app using a sampling without replacement method to 1 of 6 vignettes describing a person with obsessive-compulsive (OC) symptoms from 1 out of 6 types of content (ie, aggression or harm; sexual; religious, blasphemous, or immoral; contamination or washing; doubts or checking; or superstition, symmetry, or order). The vignettes consisted of descriptions of patients presenting with OC symptoms, with similar severity. Furthermore, interference and impairment in quality of life were described. The descriptions were based on real clinical cases [34]. All of them meet the diagnosis criteria following the *Diagnostic and Statistical Manual of Mental Disorders, Fifth Edition*, and the labels of obsessions or compulsions were avoided. All the described patients were referred to as A; sex was not specified, and they were middle aged. Furthermore, all descriptions included between 166 and 175 words.

After reading the assigned vignette, the participants were asked by the app to complete the preintervention measures. Most of them (ie, Spanish Mental Illness Stigma Attribution Questionnaire–27 [AQ-27-E)], General Help-Seeking Questionnaire [GHSQ], Social Distance Scale [SDS], and MHL questionnaire) were answered in reference to the assigned vignette. Only after completing the preassessment were the participants able to begin playing. The participants who completed the 10 missions and finished the game were asked through the app to complete the postintervention assessment and the 3-month follow-up assessment. To encourage the participants to complete the game, they were given entries to a prize draw for a voucher to spend on the web.

### Intervention Program

The esTOCma beta version is a serious game whose content and videos were developed by Doctor of Philosophy–level clinical psychologists who are experts on OCD, together with a usability expert. A professional designer developed the graphic elements, and a computer engineer developed the app. After testing multiple prototypes, this version was developed (Multimedia Appendix 1).

**Figure 1:**
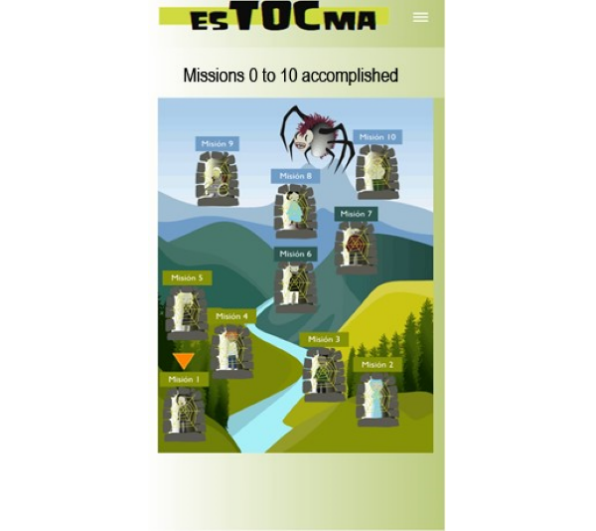
The missions, set along a mountain road.

During the game, participants are asked to fight against the OCD “stigma monster” with their knowledge by accomplishing 10 missions and freeing the 10 characters who are affected by the esTOCma monster, a creature that feeds on false beliefs and misinformation in society [35] (Figure 1). The participants are guided through the game by a woman who describes herself as an OCD expert.

The game is organized into three different intervention mechanisms: (1) psychoeducation (general information about OCD, OCD heterogeneity, OCD dimensionality, OCD cognitive model, evidence-based treatments, and options for seeking help in OCD), which includes 5 missions, and 1 of them—mission 3—includes a video explaining the OCD cognitive model (1 min); (2) indirect contact (including 6 videos of approximately 2 to 3 minutes of 3 patients diagnosed with OCD who talk about their own experience with OCD: symptom description, interference, how long it took them to seek help, their experience in disclosing that they had a disorder, and experience with psychotherapy), which is organized into 2 missions; and (3) cognitive restructuring to replace dysfunctional beliefs related to rejection toward people with OCD, variables involved in OCD development, and treatment options and effectiveness, which includes 3 missions.

The missions are organized as follows: (1) the expert describes the objective of the mission and introduces the character to be freed, (2) the expert presents activities associated with the mission (between 6 and 8 activities, mostly consisting of reading a text and answering questions about it), and (3) the freed character appears. During the game, users receive basic internet-based rewards (ie, a key to free the character), together with a message of reinforcement and a video of the character actually being freed from esTOCma. At the end of the game, there is new visual reinforcement through a video in which all the characters are freed from the monster. Moreover, users receive reinforcement through a diploma that certifies them as OCD experts. A further description of the game can be found in the study by Chaves et al [35].

### Measures

The participants completed the sociodemographic information and Obsessive-Compulsive Inventory–Revised at preassessment and an acceptability questionnaire at postassessment. All other measures were completed at the preassessment, postassessment, and 3-month follow-up assessment.

#### Sociodemographic Variables

The sociodemographic variables include gender, age, educational level, level of information and communications technology (ICT) knowledge (from 1 *little or none* to 5 *expert level*), and the question of whether they have an OCD diagnosis.

#### Acceptability Questionnaire

It consisted of 3 questions developed ad hoc to assess the acceptability of the esTOCma app. The survey included 2 questions related to usefulness (ie, “Did you find the app useful?” [from *very useful* to *not useful at all*] and “Did you learn from the app?” [from *I learned a lot* to *I learned nothing*]) and 1 question related to satisfaction with the app (ie, “Would you recommend this app to a friend?” [from *a lot* to *not at all*]). The questions were multiple-choice questions with 4 alternatives.

#### AQ-27-E Measures

This measures the social stigma associated with a vignette describing a person showing OC symptoms through 27 items rated on a Likert-type scale ranging from 1 to 9 [36,37]. It includes 9 subscales with 3 items: responsibility, pity, anger, dangerousness, fear, no help, coercion, segregation, and avoidance. Higher scores indicate higher social stigma. In this study, the AQ-27-E scales showed acceptable to excellent internal consistency across subscales (from 0.70 [anger, 3-month follow-up] to 91 [fear, 3-month follow-up]), except for responsibility (ranging from 0.40 [postassessment] to 0.63 [preassessment]) and pity (ranging from 0.36 [preassessment] to 0.52 [3-month follow-up]).

#### GHSQ Measures

This measures the intention to seek help from 10 different sources with regard to the specific content described in a vignette describing a person showing OC symptoms [38,39]. Participants rated 10 items regarding their help-seeking intentions if they were experiencing from symptoms similar to those described in the vignette on a 7-point Likert-type scale ranging from 1 (*extremely unlikely*) to 7 (*extremely likely*). Scores were calculated by summing up the items and dividing by 10. Higher scores indicate a higher intention to seek help. In this, the GHSQ showed acceptable internal consistency at the different assessment points (Cronbach α ranging from 0.68 [preassessment] to 0.75 [postassessment]).

#### SDS Measures

This assesses an individual’s willingness to interact with an individual with a mental disorder described in a vignette across 7 different situations on a 4-point Likert scale from 0 (*definitely willing*) to 3 (*definitely unwilling*) [40]. The total score has been calculated by adding the scores and dividing by the 7 items, with higher scores indicating a greater preference for social distance. In this, the SDS showed between good and excellent internal consistency at all assessment points; Cronbach α ranged from 0.86 at postassessment to 0.93 at the 3-month follow-up.

#### MHL Questionnaire

This is an instrument developed for this study based on previous studies [25,41] and assesses 1 of the components defined by Kutcher et al [42] as MHL but only referring to OCD: the understanding of OCD and its treatment. Part 1 has 4 multiple-choice questions including between 2 and 7 alternative answers, only 1 of which is correct. The questions refer to the assigned vignette (person A) and evaluate the following dimensions: (1) problem recognition (ie, “What happens to A is cause for concern?”; response alternatives: yes and no), (2) OCD identification (ie, “What do you think might be happening to A?” This question includes 7 response alternatives: family problems, adjustment problems, anxiety disorder, generalized anxiety disorder, schizophrenia, OCD, and depression), (3) perception of causality, and (4) effective treatment option. Part 2 has four multiple-choice questions with 3 alternative answers, only 1 of which is correct, and refers to participants’ general knowledge of OCD: (1) identification of OCD as a mental disorder (ie, OCD is [a] a learning disorder, [b] a mental disorder, or [c] a set of manias); (2) definition of obsession; (3) definition of a compulsion; and (4) role played by compulsions and other control strategies in the maintenance of obsessions. A total score has been calculated as the sum of the correct answers, thus ranging from 0 to 8.

#### Obsessive-Compulsive Inventory–Revised Measures

This is an 18-item self-report questionnaire assessing distress caused by OC symptoms and rated on a 5-point Likert scale ranging from 0 (not at all) to 4 (extremely) [43,44]. A total score was calculated. The total score of the Obsessive-Compulsive Inventory-Revised showed excellent internal consistency at all assessment points in this study (Cronbach α ranging from 0.89 [postassessment] to 0.90 [preassessment]).

### Statistical Analysis

Descriptive statistics (eg, means, SD, and frequencies or percentages) were used to analyze demographic data, study variables, and app use patterns. Chi-square and 1-tailed *t* tests were used to test whether the groups (completers vs noncompleters) had preexisting differences. The change in study variables over time was determined by a repeated measures ANOVA. Partial eta–squared was used to report the effect size of the intervention on the dependent measures. A mixed model was used to determine whether the pattern of use of the app affected its effectiveness. The within-participants factor was time, and the between-participants factor was whether the user followed the 1 mission per day recommendation. The statistical significance level was set at *P*=.05. SPSS Statistics (version 26; IBM Corp) was used for statistical analysis.

### Ethical Considerations

All procedures described in the study have been approved by the Human Research Ethics Committee of the University of Valencia, Spain (1276901). All study participants provided informed consent before study enrollment.

## Results

### Feasibility

#### Recruitment

**Figure 2:**
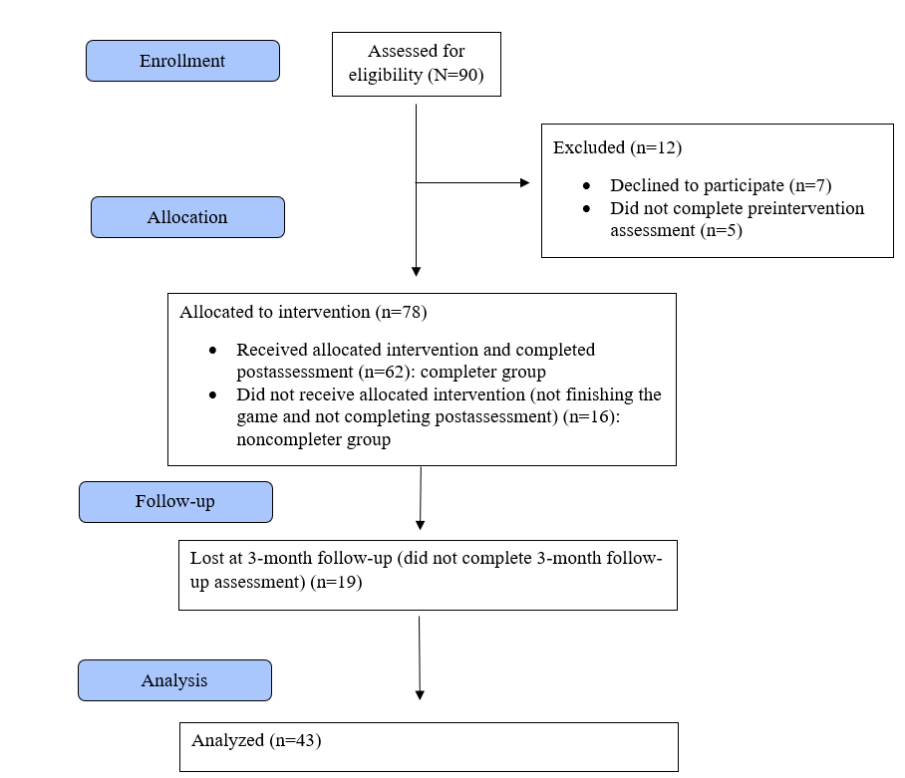
Participants’ flowchart.

A total of 90 participants were enrolled in the study; they downloaded the app and met the inclusion criteria. Of these 90 participants, 86% (78/90) were allocated to intervention and 13% (12/90) were excluded as they did not provide informed consent or did not complete the preassessment intervention (Figure 2). Of these 78 participants, 20% (16/78) did not complete the game or the postassessment (noncompleter group) and 79% (62/78) completed the game and answered the postintervention assessment (completer group). In the completer group, 69% (43/62) of the participants completed the 3-month follow-up assessment, and the remaining 30% (19/43) of the participants were lost at the 3-month follow-up.

#### Participants’ Characteristics

The completer group had a mean age of 36.74 years, ranging from 18 to 71 years; were mostly women; and had university studies and moderate ICT knowledge. Differences in sociodemographic characteristics and preintervention assessment (baseline) between the completer group and the noncompleter group were calculated (Table 1).

**Table 1:** Differences between the completers and noncompleters on demographic and study variables (N=90).

Variable or measure			Completers (n=62)		Noncompleters (n=16)		Chi-square (*df*)		*t* test (*df*)		*P* value
Age (y), mean (SD)			36.74 (14.41)		27.19 (9.26)		10.4 (2)		N/A^a^		.003
Gender (women), n (%)			38 (61)		7 (44)		2.0 (2)		N/A		.35
**Education level, n (%)**							2.2 (2)		N/A		.31
	Primary	3 (5)		0 (0)							
	Secondary	11 (18)		1 (6)							
	University	48 (77)		15 (94)							
**Knowledge of ICT^b^, n (%)**							2.8 (2)		N/A		.58
	Little or none	1 (2)		0 (0)							
	Low	5 (8)		0 (0)							
	Moderate	35 (57)		10 (63)							
	Advanced	18 (29)		4 (25)							
	Expert level	3 (5)		2 (13)							
MHL^c^ (total^d^), mean (SD)			6.79 (1.50)		5.93 (1.61)		N/A		1.992 (76, 36.104)		.05
AQ-27-E^e^ (total^f^), mean (SD)			84.77 (24.81)		77.25 (25.52)		N/A		1.075 (76, 36.104)		.28
SDS^g^ (total^h^), mean (SD)			1.00 (0.64)		0.78 (0.70)		N/A		1.207 (76, 36.104)		.23
GSHQ^i^ (total^j^), mean (SD)			4.20 (0.92)		4.27 (0.83)		N/A		0.308 (76, 36.104)		.38
OCI-R^k^ (total^l^), mean (SD)			18.17 (13.23)		20.06 (10.70)		N/A		0.526 (76, 36.104)		.60

^a^N/A: not applicable.

^b^ICT: information and communications technology.

^c^MHL: Mental Health Literacy Questionnaire.

^d^Total score ranging from 0 to 8.

^e^AQ-27-E: Spanish Mental Illness Stigma Attribution Questionnaire–27.

^f^Total scoring from 27 to 243.

^g^SDS: Social Distance Scale.

^h^Total score ranging from 0 to 3.

^i^GHSQ: General Help-Seeking Questionnaire.

^j^Total scoring from 1 to 7.

^k^OCI-R: Obsessive-Compulsive Inventory–Revised.

^l^Total scoring from 0 to 72.

Statistically significant differences were only observed in age, with the completer group being older, and MHL scores, which were higher in the completer group. In addition, in the completer group, we explored the differences between the participants who completed the 3-month assessment (43/62, 69%) and those who did not (19/62, 31%). The only discernible difference observed was in age (t_60_=2.167; *P*=.01), with participants who completed the follow-up assessment being older.

Of the 43 participants included in the effectiveness analyses, most were women (n=28, 65%) with a mean age of 39.30 (SD 14.58; range 21-71) years, with university-level education (n=35, 81%), and with ICT knowledge between moderate (n=23, 54%) and advanced (n=15, 35%; only 3 (7%) participants described having between little or none and low ICT knowledge level).

#### App Use Pattern

An analysis of the app use pattern was conducted with the completer group. First, we explored the number of days that elapsed from the preassessment to the postassessment. The participants took a mean of 13.64 (SD 10.50; range 1-44) days to complete the app (which includes having done the preassessment, the 10 missions and the postassessment), with a mode of 1, that is, the most frequent pattern was conducting the pre- and postassessments and missions of the app in 1 day. Second, we analyzed the number of days the person spent performing the missions. The participants spent between 1 and 10 days performing the missions, with a mean of 4.56 (SD 3.33) days and a mode of 1. In total, 30% (19/62) of the participants completed the 10 missions in 1 day, whereas another 21% (13/62) of the participants spent between 9 and 10 days completing the app, that is, approximately 1 session per day.

Finally, we explored the minutes spent performing each mission, first excluding the participants who stayed on 1 mission for >20 minutes, as we assumed that they left the app open without using it. The data on the participants completing the missions in <20 minutes is presented in Table 2.

**Table 2:** Time (in min) spent in completing each of the 10 missions for participants who complete the mission in <20 minutes.

	Participants, n (%)^a^	Time (min), mean (SD; range)	Mode (min)
Mission 1	58 (94)	3.93 (1.02; 1-6)	4
Mission 2	54 (87)	4.44 (1.90; 2-10)	4
Mission 3	35 (56)	3.80 (1.37; 1-8)	3
Mission 4	35 (56)	2.06 (1.64; 0-10)	2
Mission 5	35 (56)	5 (3.11; 2-20)	4
Mission 6	56 (96)	8.71 (1.78; 7-20)	8
Mission 7	29 (47)	9.35 (3.06; 7-20)	8
Mission 8	33 (53)	3.85 (1.48; 2-8)	3
Mission 9	58 (94)	2 (3.01; 0-19)	0
Mission 10	56 (90)	2.09 (2.26; 0-9)	0

^a^n (%) of participants completing the mission in ≤20 minutes.

The data are displayed in minutes.

Between the participants who completed missions in ≤20 minutes, and considering the mode, missions included in module 1 (ie, missions 1 to 5) took between 2 and 4 minutes, in module 2 (ie, missions 6 to 7) took 8 minutes, and in module 3 (ie, missions 8 to 10) took between <1 minute and 3 minutes.

### Acceptability

After completing the app until the end of the game, most participants (56/62, 90%) perceived the app as useful or very useful. Moreover, 90% (56/62) of the participants indicated that they had learned or learned a lot, and 98% (61/62) of the participants indicated that they would recommend the app to a friend.

### Preliminary Effectiveness: Differences Between Pre- and Postintervention Assessments and 3-Month Follow-Up

Repeated measures ANOVA was conducted to examine differences between pre- and postintervention and the 3-month follow-up on knowledge of OCD and its treatments (MHL questionnaire), stigmatizing attitudes (AQ-27-E), social distance (SDS), and intention to seek help (GHSQ) associated with OCD (Table 3). The results showed statistically significant differences (*P*≤.05) in all variables with medium-to-large effect sizes, except for the second part of the MHL questionnaire and the pity and coercion subscales (AQ-27-E). In general, the results show that using esTOCma until the end of the game results in an increase in MHL and intention to seek treatment (GHSQ) and a decrease in stigmatizing attitudes (AQ-27-E) and social distance desire (SDS). Post hoc pairwise comparisons showed statistically significant differences between preintervention and the other 2 assessment points (postintervention and 3-month follow-up). No significant differences were observed between postintervention and the 3-month follow-up in the variables assessed, except for the MHL total score, in which follow-up scores did not differ from pre- to postintervention.

**Table 3:** Means (SDs) and repeated measures ANOVA on pre-, post-, and 3-month follow-up intervention scores (n=43).

Variable or measure		Pretreatment, mean (SD)	Posttreatment, mean (SD)			3 month follow-up, mean (SD)		*F* test (*df*)^a^		*P* value		*η_*p*_^2b^*
**MHL^c^**												
	Part 1^d^	3.32 (0.80)^e^		3.72 (0.54)^e^	3.62 (0.61)^e^		6.523 (1.566, 65.769)		.005		0.134	
	Part 2^f^	3.58 (0.69)		3.72 (0.50)	3.77 (0.57)		1.896 (1.513, 63.526)		.16		0.043	
	Total score	6.90 (1.34)^e^		7.44 (0.93)^e^	7.39 (0.90)^e^		5.754 (1.325, 55.662)		.01		0.120	
**AQ-27-E^g^**												
	Responsibility	9.04 (4.07)^e^		6.97 (3.70)^e^	7.30 (4.35)^e^		8.103 (2, 84)		.001		0.162	
	Pity	17.30 (4.15)		18.62 (4.36)	18.20 (4.68)		1.984 (2, 84)		.14		0.045	
	Anger	8.23 (4.44)^e^		5.81 (3.42)^e^	5.76 (3.19)^e^		10.554 (1.614, 67.813)		<.001		0.201	
	Dangerousness	7.11 (4.31)^e^		4.72 (2.65)^e^	5.37 (4.01)^e^		7.386 (2, 84)		<.001		0.150	
	Fear	6.39 (4.26)^e^		5.00 (3.72)^e^	4.23 (2.42)^e^		6.489 (2, 84)		.002		0.134	
	No help	8.09 (4.68)^e^		5.69 (3.32)^e^	5.83 (4.05)^e^		9.320 (2, 84)		<.001		0.182	
	Coercion	12.00 (5.30)		11.18 (5.50)	10.93 (6.47)		0.983 (1.490, 62.601)		.35		0.023	
	Segregation	5.16 (3.92)^e^		4.00 (1.96)^e^	4.04 (2.22)^e^		4.762 (1.555, 65.297)		.01		0.102	
	Avoidance	11.88 (5.66)^e^		8.58 (6.35)^e^	7.76 (5.48)^e^		16.938 (2, 84)		<.001		0.287	
SDS^h^		0.99 (0.58)^e^		0.64 (0.65)^e^	0.59 (0.63)^e^		10.597 (2, 84)		<.001		0.201	
GSHQ^i^		4.33 (0.84)^e^		4.84 (0.86)^e^	4.58 (1.03)^e^		6.818 (2, 84)		.002		0.140	

^a^Dfs were Greenhouse-Geisser corrected where appropriate.

^b^*ηp^2^*:partial eta squared for within-subject contrasts (ANOVA).

^c^MHL: Mental Health Literacy Questionnaire, total score ranging from 0 to 8.

^d^Part 1 scoring from 0 to 4.

^e^Significant differences among groups (*P*≤.05).

^f^Part 2 scoring from 0 to 4.

^g^AQ-27-E: Spanish Mental Illness Stigma Attribution Questionnaire–27; subscales ranging from 3 to 27.

^h^SDS: Social Distance Scale, ranging from 0 to 3.

^i^GHSQ: General Help-Seeking Questionnaire, scoring from 1 to 7.

Finally, we examined whether the pattern of use of the app influenced the effectiveness of the intervention with a 2 (group: individuals who follow the recommendation of 1 mission per day [ie, 9/10, % days] [12/N, %; percentile 75] vs individuals who did it in another way [31/N, %])×3 (time: pre-, post-, and follow-up assessments) repeated measures mixed ANOVA. The results show that there was no significant group×time interaction (*F*_26,16_=0.838; *P*=.67). Univariate follow-up analyses also indicated no significant group×time effect for any of the measured variables (*P*>.05).

## Discussion

### Principal Findings

This study is the first to investigate the feasibility, acceptability, and effectiveness of a mobile health app designed to enhance several variables related to OCD, which were identified by Kutcher et al [42] as components of MHL: understanding OCD and its treatments, decreasing stigma associated with OCD, and enhancing help-seeking effectiveness. Our findings show that esTOCma was feasible and acceptable and that after using it until game completion, there was a positive change in the variables of interest, which lasted for at least 3 months.

Of the participants allocated to the intervention, approximately 80% (78/90) completed the app, and of the who completed the intervention, approximately 70% (62/78) completed the follow-up assessment. This adherence rate is similar to or higher than that reported in other internet-based studies with self-help interventions, with dropouts being one of the main challenges of interventions with mental health apps [45-47].

The participants who completed the study were older and had higher knowledge of OCD than those who began the study but did not complete it. However, there were no differences in participants’ knowledge of new technologies, which suggests that the app is easy to use and does not require a significant amount of knowledge to become involved in its use, although >50% (35/62) of the participants described themselves as having moderate ICT knowledge. Regarding the pattern of use, although it was recommended that participants complete 1 mission per day and participants were reminded of this recommendation after finishing each mission, the most common pattern of use was to complete the app, including pre- and postevaluations, in 1 day. In fact, only 21% (13/62) of the participants completing the intervention followed the recommendation to perform 1 mission per day. It seems that it is more comfortable for participants to perform more missions per day; otherwise, they forget to complete the mission the following day. In fact, the participants completed their participation in the study within a range of 1 to 44 days.

Regarding the time invested in each mission, although missions were quick to complete and always took <10 minutes, the participants often left missions midway and continued later the same day or days later. Furthermore, certain missions were abandoned in the middle more frequently, suggesting that they could be reformulated to make them more “attractive.” This was the case for mission 7, which was interrupted by >50% (31/62) of the participants, as well as for missions 3, 4, 5, and 8. The pattern of use and interruptions does not seem to be associated with the intervention mechanism on which the mission is based but perhaps with the content or the duration of the mission. If we analyze missions 1, 2, 6, 9, and 10, those that were carried out without interruption, we see that missions 1 and 2 are the initial ones and deal with content describing obsessions and compulsions; mission 6 includes the first videos that also describe symptomatology and interference, without additional theoretical content to read; and missions 9 and 10 (cognitive restructuring) are characterized by being very brief and with less theoretical content than missions 1 to 5 (psychoeducation). It seems that the description of symptomatology, as well as more dynamic and shorter missions, result in more attractive missions or at least in missions that capture participants’ attention to a greater extent.

In general, the participants seemed satisfied with the app, as approximately 100% (62/62) would recommend it to a friend and >90% (56/62) consider it useful and that they have learned about OCD.

Regarding the effectiveness of the app, the results show an increase in OCD knowledge. Our data match those of the previous interventions that have found increases in general knowledge of OCD after offering written information about the disorder [25,26]. The data are also consistent with other technology interventions that have increased MHL levels on different mental health problems [48-50]. However, the differences were not statistically significant in those questions that asked about OCD in general (eg, the definition of an obsession), that is, not referring to the description of a person showing OC symptoms. This could be due to a ceiling effect, as the scores were already high in the preassessment evaluation. In fact, they were higher among those participants who played with the app until the game was over in comparison with those who did not finish the game. In this sense, our first hypothesis was only partially supported.

Regarding the hypothesized decrease in stigmatizing attitudes, the results support our hypothesis, as they suggest that after completing the 10 missions of the app, there was a decrease in some stereotypes or public attitudes, such as the perception of OC symptoms as dangerous; emotional reactions of anger or fear toward people showing OC symptoms; discriminating behaviors such as the intention of not helping, segregating, or avoiding people showing OC symptoms; or the desire to maintain social distance. Although a video-based intervention decreased social distance desire [29] and 2 interventions centered on reading the Diagnostic and Statistical Manual of Mental Disorders, Fifth Edition diagnostic criteria for OCD decreased negative attitudes about violent and sexual thoughts [28,51], other interventions consisting of reading information on OCD (psychoeducation) showed small changes in stigmatizing attitudes [25]. The results suggest that the esTOCma intervention, which includes psychoeducation but also incorporates components such as contact, seems to change stigmatizing attitudes to a greater extent, with medium-to-large effect sizes.

Our data are also consistent with previous research also using new technologies to reduce stigma associated with other mental disorders that have reported a decrease in dangerousness, anger, fear, segregation, and avoidance [15,52,53]; a decrease in stigma as a general measure [53-55]; an increase in the help factor [52]; or a decrease in social distance [52,56]. This is a remarkable result, as traditional antistigma interventions not using innovative technologies often report small-to-medium effect sizes [14,52]. Moreover, the results are based on a community sample with an average age higher than those used in studies reporting interventions using innovative technologies [16], suggesting that these types of interventions could also be useful for older people.

In addition to the changes in stigmatizing attitudes after using esTOCma, there were no significant changes in 2 of the social stigma dimensions measured by the AQ-27-E: pity and coercion. Previous studies have also reported a lack of changes in pity [57]. In fact, it has been suggested that pity should be considered, at least in some contexts, not to be a factor of stigma but rather a reflection of compassion and the capacity to empathize with people with mental health problems [58], and research has shown associations between pity and the tendency to help [37]. Regarding coercion, there is no significant change after the intervention in the belief that people with OCD should receive treatment, even if they refuse it (coercion). This is consistent with previous interventions in the OCD field [25].

Finally, the results also support our third hypothesis, as there was a large effect size improvement in the intention to seek help when experiencing symptoms similar to those represented in the different vignettes. Thus, increasing knowledge of OCD and its treatment could have acted as a help-seeking facilitator [59,60]. To the best of our knowledge, there are no interventions to improve help seeking associated with OCD, and thus, our results are of great relevance, as research shows that early help seeking is associated with a better treatment response, earlier remission of symptoms, and recovery from the disorder [9,61]. The data are consistent with other studies that, through mental health apps [62] and other technology-based interventions, have improved the intention to seek help for other mental health problems [63].

Moreover, our study showed a maintained effect in the study variables at the 3-month follow-up assessment. This is especially relevant as most of the interventions do not include a follow-up assessment [16,50,63,64], and only 1 intervention on OCD has included a follow-up assessment [29].

### Limitations and Recommendations

This study has limitations. As a pilot study, the study sample was small and did not include a control group. There was also a considerable dropout rate that could be associated with the fact that participants forgot to complete the app (to do all 10 missions) as well as the large number of assessment questionnaires included. Furthermore, considering that OCD is a heterogeneous disorder and that there are differences in stigma and OCD recognition between different types of content [41,64,65], we decided to randomize participants to 6 vignettes that represent OCD heterogeneity. However, this decision could be considered as a limitation of the design of this study that could affect the effectiveness of the data.

Despite these limitations, our results provide valuable information for a broader implementation of the app. First, as future lines of work, we propose making an effort to motivate participants, given that higher adherence was associated with initial knowledge of OCD or sensitivity toward mental health problems. Second, a potential strategy to attract a younger audience, as adherence was also associated with older age, could involve transforming the app into a serious game, aligning esTOCma with the immersive experience of video games. Actively engaging participants through gamification elements could further contribute to increasing adherence. Looking ahead, evolving esTOCma into a serious game with an adventure game theme holds promise for future developments and for addressing the stigma associated with OCD. Third, pop-ups and emails to remind participants to “play” could be incorporated, as those participants who completed the participation took up to 44 days to finish an app recommended to be conducted in 10 days. Fourth, given that the number of missions per day seems not to influence the effectiveness of the app, the recommendation to play 1 mission per day could be omitted, as this is not the preferred pattern of use of participants. Finally, given that some missions were completed with interruptions by approximately half of the participants, it would be recommended to inform users of how long each mission will last. Furthermore, missions 3 to 5, 7, and 8 should be revised, as although they were quick to complete (between 3 and 8 min), the participants often left them midway and continued later. They could perhaps be shortened or made more dynamic.

### Future Research

The results suggest the potential for the use of this intervention app and provide the basis for developing a larger randomized controlled study to validate the use of esTOCma (version 1.0), as has been proposed [35]. Importantly, we will also explore differences in OCD types of content through a controlled study, as research shows that there are differences in OCD recognition and stigma considering the different OCD types of content [41,64,65]. Furthermore, it will be of interest to explore the effectiveness of each intervention mechanism (ie, psychoeducation, indirect contact, and cognitive restructuring) to understand whether there are differences between them. This analysis would allow us to develop a new app that would eliminate those mechanisms that are less effective or improve them. Future studies should also examine the usefulness of this app in OCD cohorts, especially in the first stages of diagnosis, and their families. Through esTOCma, people with OCD could receive (or reinforce) cognitive-behavioral psychoeducation and be assisted in their search for empirical-based treatments. Furthermore, esTOCma could eliminate self-stigma, improving their quality of life. In terms of family involvement, esTOCma could assist them in comprehending and managing OCD symptoms as well as support their relatives with OCD in seeking effective treatment.

### Conclusions

Our findings show that esTOCma is a feasible and acceptable app and that after completing its 10 missions, there is an increase in the understanding of OCD and help-seeking intention as well as a decrease in the social stigma and social distance associated with OCD that lasts for at least 3 months. These changes might result in less delay in seeking help and a better treatment response to the problem and prognosis. Moreover, providing mental health knowledge to the community population as to the nature and universality of intrusive thoughts may protect and prevent the general population from developing OCD and, furthermore, reduce the economic and personal costs associated with OCD.

## Appendix

Multimedia Appendix 1 Screenshots of the esTOCma app with examples of the Psychoeducation mechanism.

## Abbreviations

AQ-27-E: Spanish Mental Illness Stigma Attribution Questionnaire–27
GHSQ: General Help-Seeking Questionnaire
ICT: information and communications technology
MHL: mental health literacy
OC: obsessive-compulsive
OCD: obsessive-compulsive disorder
SDS: Social Distance Scale
