# Supplementary material for: The Effectiveness of a Cell Phone eHealth App in Changing Knowledge, Stigmatizing Attitudes, and Intention to Seek Help Associated With Obsessive-Compulsive Disorder: Pilot Questionnaire Study
Source: JMIR Mhealth Uhealth. 2024 Mar 29;12:e48027. doi: 10.2196/48027 (PMC11015362; doi:10.2196/48027)
Supplement: Multimedia Appendix 1 [file mhealth_v12i1e48027_app1.pdf]

Screenshots of the esTOCma app with examples of the Psychoeducation mechanism.

Supplementary material:

Chaves, A., Arnáez, S. & García Soriano, G. (2024). Effectiveness of a cell phone e-health application in changing knowledge, stigmatizing attitudes, and intention to seek help associated with obsessive-compulsive disorder: a pilot study. JMIR Mhealth Uhealth. doi:10.2196/48027
http://dx.doi.org/10.2196/48027

Screenshot from the first screen.

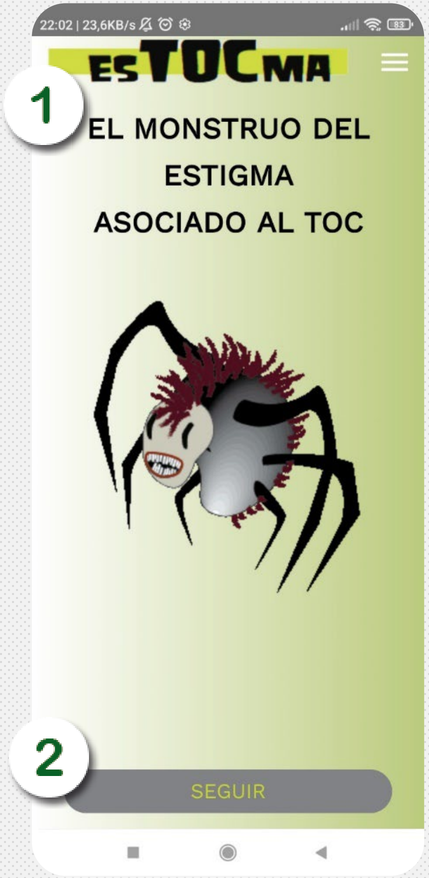

1 OCD STIGMA MONSTER

2 CONTINUE

Screenshot from a video explaining the OCD cognitive model (Mission 3).

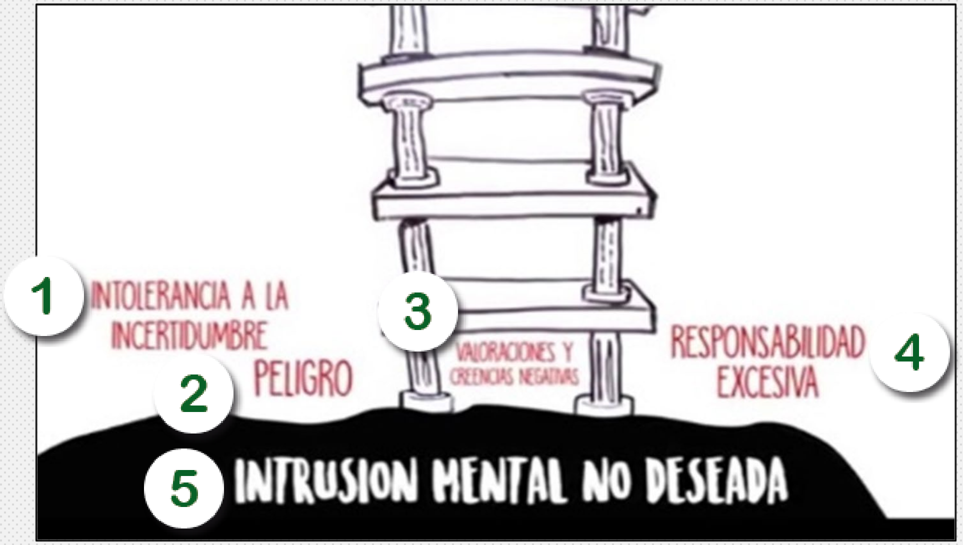

1. Intolerance to uncertainty
2. Danger
3. Appraisals and dysfunctional beliefs
4. Overestimation of responsibility
5. Unwanted mental intrusion

Screenshot with an example of the questions about the cognitive model (Mission 3).

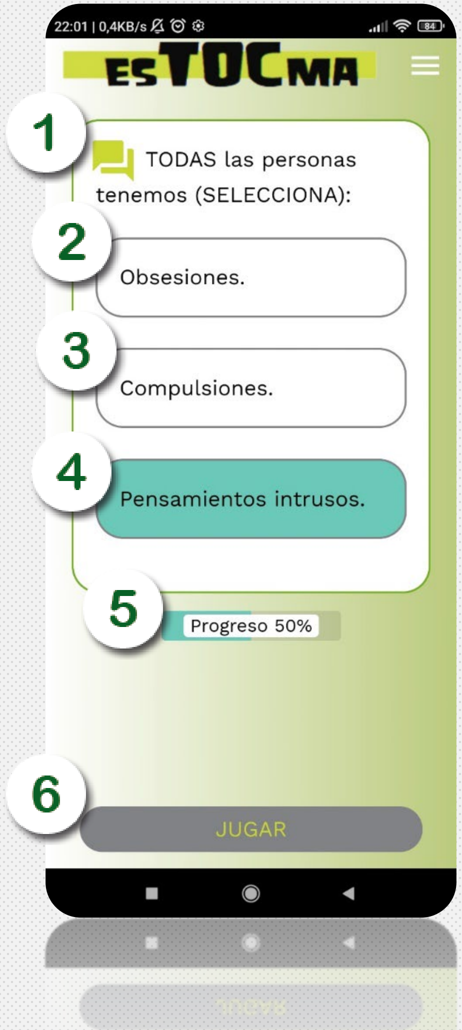

1 ALL people have (SELECT):

2 Obsessions.

3 Compulsions.

4 Intrusive thoughts

5 Progress 50%

6 PLAY

Screenshot with an example of the questions about the cognitive model (Mission 3).

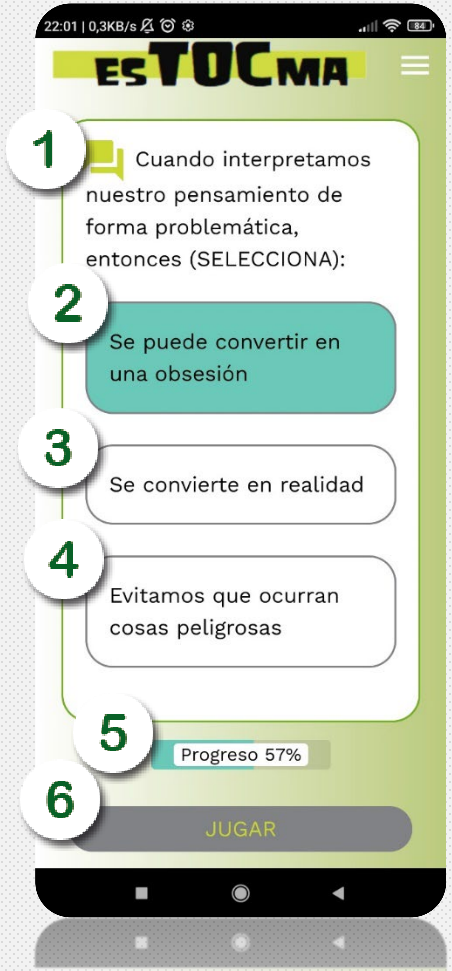

1. When we interpret our thought in a problematic way, then (SELECT):
2. It can become an obsession.
3. It becomes real.
4. We prevent dangerous things from happening.
5. Progress 57%
6. PLAY

Screenshot with Mission 4 unlocked to begin playing.

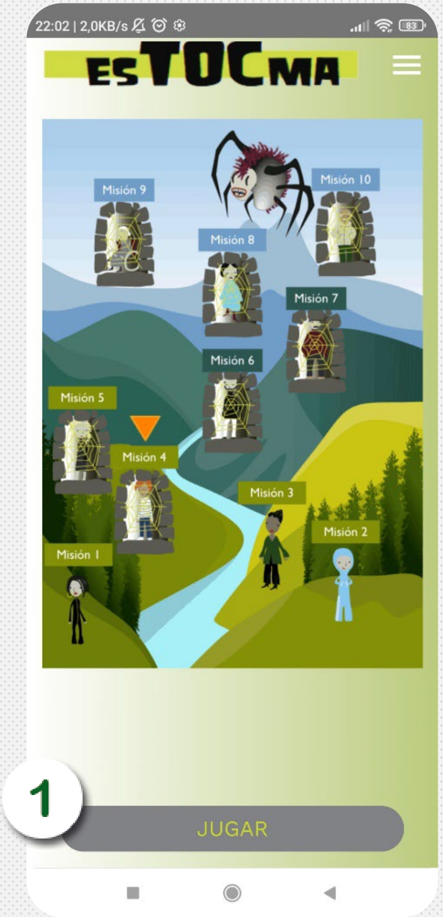

1 PLAY

Screenshot in which the OCD expert presents the objectives of Mission 4.

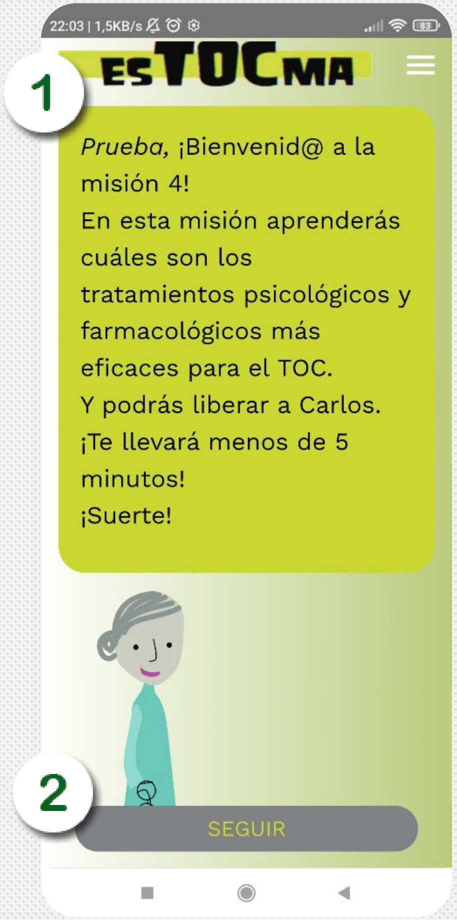

1 Nickname, Welcome to mission 4!
In this mission you will learn which are the most effective psychological and pharmacological treatments for OCD.
And you will be able to free Carlos.
It will take you less than 5 minutes!
Good luck!

2 CONTINUE

Screenshot presenting Carlos, the character to be freed in Mission 4.

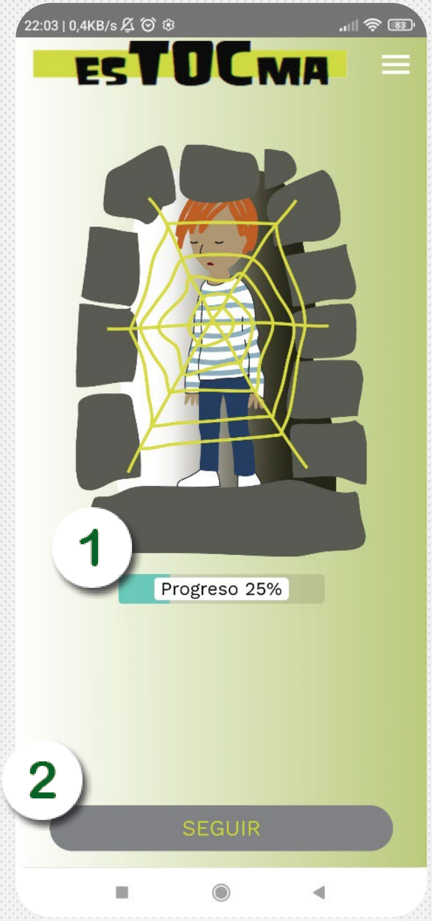

1 Progress 25%

2 CONTINUE

Screenshot from the Key fact section at the end of Mission 4.

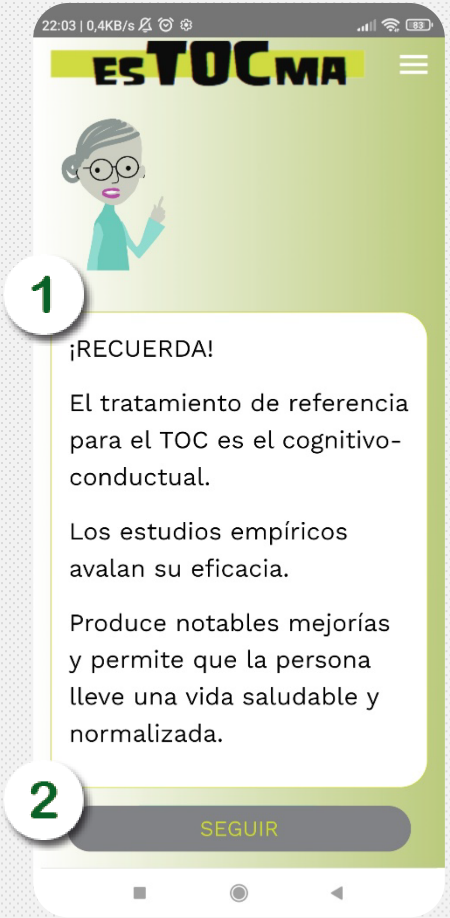

1

REMEMBER!
The gold standard treatment for OCD is cognitive-behavioral.
Empirical studies support its efficacy.
It produces remarkable improvements and allows the person to lead a healthy and normalized life.

2 CONTINUE

Screenshot from a freed character (Mission 5).

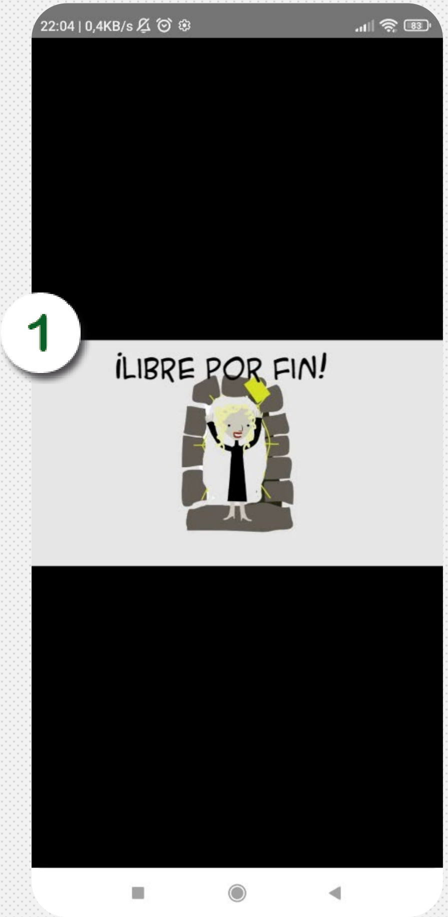

1 FINALLY FREE!

Screenshot from the contextual menu: Key facts section.

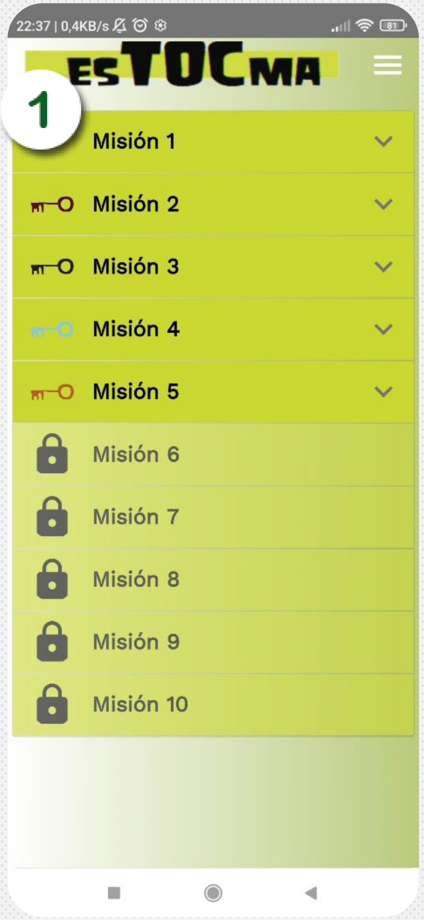

1 Mission 1
